# Supplementary material for: The DNA adenine methylase of Salmonella Enteritidis promotes their intracellular replication by inhibiting arachidonic acid metabolism pathway in macrophages
Source: Front Microbiol. 2023 Mar 2;14:1080851. doi: 10.3389/fmicb.2023.1080851 (PMC10018194; doi:10.3389/fmicb.2023.1080851)
Supplement: Supplementary file 2 [file Data_Sheet_2.zip › S1 Appendix. Non-targeted metabolomics raw data/4.MetDiffAnalysis/C50336_Ddam.vs.control/C50336_Ddam.vs.control_neg_heatmap2.pdf]

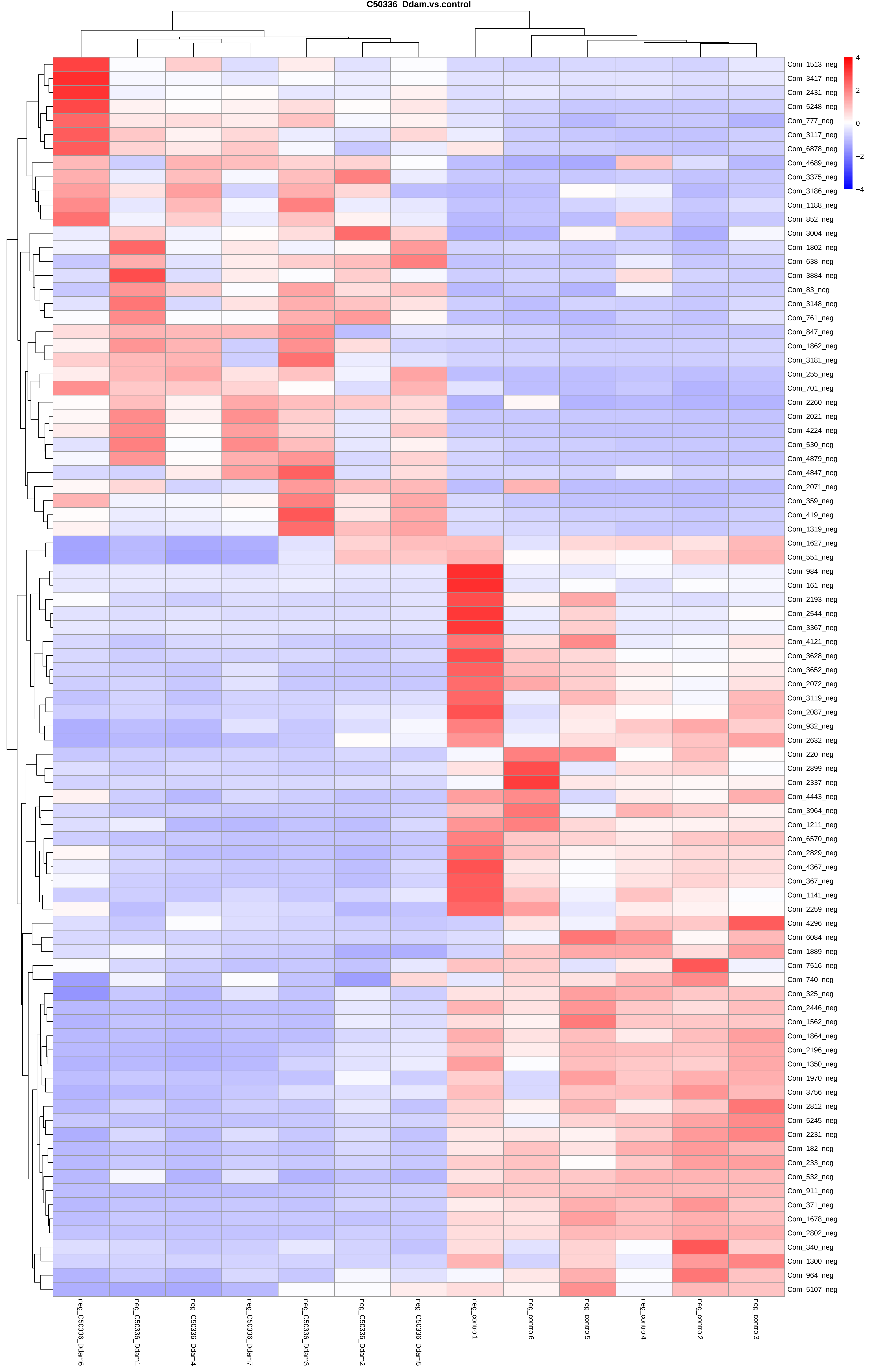

C50336_Ddam.vs.control
4
2
0
−2
−4
Com_1513_neg
Com_3417_neg
Com_2431_neg
Com_5248_neg
Com_777_neg
Com_3117_neg
Com_6878_neg
Com_4689_neg
Com_3375_neg
Com_3186_neg
Com_1188_neg
Com_852_neg
Com_3004_neg
Com_1802_neg
Com_638_neg
Com_3884_neg
Com_83_neg
Com_3148_neg
Com_761_neg
Com_847_neg
Com_1862_neg
Com_3181_neg
Com_255_neg
Com_701_neg
Com_2260_neg
Com_2021_neg
Com_4224_neg
Com_530_neg
Com_4879_neg
Com_4847_neg
Com_2071_neg
Com_359_neg
Com_419_neg
Com_1319_neg
Com_1627_neg
Com_551_neg
Com_984_neg
Com_161_neg
Com_2193_neg
Com_2544_neg
Com_3367_neg
Com_4121_neg
Com_3628_neg
Com_3652_neg
Com_2072_neg
Com_3119_neg
Com_2087_neg
Com_932_neg
Com_2632_neg
Com_220_neg
Com_2899_neg
Com_2337_neg
Com_4443_neg
Com_3964_neg
Com_1211_neg
Com_6570_neg
Com_2829_neg
Com_4367_neg
Com_367_neg
Com_1141_neg
Com_2259_neg
Com_4296_neg
Com_6084_neg
Com_1889_neg
Com_7516_neg
Com_740_neg
Com_325_neg
Com_2446_neg
Com_1562_neg
Com_1864_neg
Com_2196_neg
Com_1350_neg
Com_1970_neg
Com_3756_neg
Com_2812_neg
Com_5245_neg
Com_2231_neg
Com_182_neg
Com_233_neg
Com_532_neg
Com_911_neg
Com_371_neg
Com_1678_neg
Com_2802_neg
Com_340_neg
Com_1300_neg
Com_964_neg
Com_5107_neg
neg_C50336_Ddam6
neg_C50336_Ddam1
neg_C50336_Ddam4
neg_C50336_Ddam7
neg_C50336_Ddam3
neg_C50336_Ddam2
neg_C50336_Ddam5
neg_control1
neg_control6
neg_control5
neg_control4
neg_control2
neg_control3
